# The urotensin II receptor antagonist DS37001789 ameliorates mortality in pressure-overload mice with heart failure

**DOI:** 10.1016/j.heliyon.2020.e03352

**Published:** 2020-02-03

**Authors:** Mina Nishi, Hideki Tagawa, Masumi Ueno, Shinji Marumoto, Takahiro Nagayama

**Affiliations:** a Specialty Medicine Research Laboratories II, Daiichi-Sankyo Co., Ltd., 1-2-58 Hiromachi, Shinagawa-ku, Tokyo 140-8710, Japan; b Daiichi Sankyo Pharma Development, Daiichi-Sankyo, Inc., 211 Mt. Airy Road, Basking Ridge, NJ 07920, USA; c Specialty Medicine Research Laboratories I, Daiichi-Sankyo Co., Ltd., 1-2-58 Hiromachi, Shinagawa-ku, Tokyo 140-8710, Japan; d Organic Synthesis Department, Daiichi-Sankyo RD Novare Co., Ltd., 1-16-13 Kitakasai, Edogawa-ku, Tokyo 134-8630, Japan; e Business Development & Licensing Department, Daiichi-Sankyo Co., Ltd., 3-5-1 Nihombashihoncho, Chuo-ku, Tokyo 103-8426, Japan

**Keywords:** Urotensin II, GPR14, Heart failure, Transverse aortic constriction, Biological sciences, Endocrinology, Diabetes, Health sciences, Cardiology, Cardiovascular system, Renal system, Pharmaceutical science, Biochemistry, Medicine, Physiology

## Abstract

This study was designed to evaluate the effects of DS37001789, a novel and highly potent urotensin II (U-II) receptor (GPR14) antagonist, against mortality, hypertrophy, and cardiac dysfunction in pressure-overload hypertrophy by transverse aortic constriction (TAC) in mice. In addition, we analyzed the phenotype of GPR14 knockout (KO) mice after TAC induction to confirm the contribution of the U-II/GPR14 system. The oral administration of 0.2% DS37001789 to TAC mice for 12 weeks significantly ameliorated the mortality rate and 0.2% DS37001789 for 4 weeks significantly improved cardiac function by pressure-volume analysis. GPR14 expression was significantly upregulated in the left ventricle in the TAC mice treated with 0.2% DS37001789. Moreover, we confirmed that the significant amelioration of mortality was accomplished by the inhibition of cardiac enlargement and the improvement of cardiac function in GPR14 KO mice after TAC surgery. These results suggest that the U-II/GPR14 system contributes to the progression of heart failure and its blockade ameliorates the mortality via improved cardiac function. The U-II/GPR14 system may thus be an attractive target for treating heart failure with pathological cardiac hypertrophy and DS37001789 may be a novel therapeutic agent for heart failure in patients with pressure-overload conditions such as hypertension and aortic valve stenosis.

## 1 Introduction

Cardiovascular disease is the major cause of death globally. The number of such patients is still increasing and expected to increase further with the aging of societies around the world. Although the advent of β-blockers, angiotensin-converting enzyme inhibitors, and angiotensin II receptor blockers has improved the prognosis of cardiovascular disease, the 5-year survival rate is still extremely poor at less than 50% and there is thus a need to develop further treatments [1].

Urotensin II (U-II) is an 11-amino-acid cyclic neuropeptide and is produced by the cleavage of prepro-U-II [2, 3]. The action of U-II is mediated by the activity of a G-protein-coupled receptor, the U-II receptor also known as GPR14 [4]. U-II and GPR14 exist in the cardiovascular region and U-II exerts strong vasoconstrictive and endothelium-dependent vasodilatory effects [3, 5]. U-II also contributes to cardiac contractility, cardiomyocyte hypertrophy, fibrosis, and inflammation [6]. In addition, the expression of U-II in the plasma and that of GPR14 in the heart were found to be increased in patients with end-stage congestive heart failure [7, 8]. These identified increases in U-II and GPR14 in patients with heart failure were further supported by several studies on chronic heart failure patients [8, 9, 10, 11]. Thus, blockade of the U-II/GPR14 system could potentially be an important therapeutic target in the treatment of heart failure. As such, the U-II/GPR14 system is considered as a promising pharmacological target in cardiovascular disease and a number of U-II receptor antagonists have been developed [12]. Although the use of some non-peptide U-II receptor antagonists has shown that blockade of the U-II/GPR14 system attenuated cardiac hypertrophy and improved cardiac function in animal models [12, 13, 14], such antagonists lack potency and have not achieved effects on mortality in heart failure animal models [14, 15]. Therefore, the effects of U-II/GPR14 system blockade on heart failure are still unclear and more potent U-II receptor antagonists are needed.

Recently, we discovered 1-{4-[2-(4-acetylpiperazin-1-yl) ethanesulfonyl]-2-[(pyrrolidin-1-yl) methyl] piperazin-1-yl-2-(2,4,5-trichlorophenoxy)ethan-1-one (DS37001789), a novel U-II receptor antagonist, which potently binds to GPR14 and also has a preferable pharmacological profile to ameliorate U-II-related pathophysiological changes in vivo [16].

To clarify the role of U-II/GPR14 system blockade in heart failure, we evaluated the effects of DS37001789 against mortality, hypertrophy, and cardiac dysfunction in pressure-overload hypertrophy using transverse aortic constriction mice (TAC). We also analyzed the phenotype of GPR14 knockout (KO) mice in the TAC model to confirm the pharmacological mechanism of DS37001789.

## 2 Materials and methods

### 2.1 Animals

Male C57BL/6 N mice (7 weeks of age) and male Sparague-Dawley rats (8 weeks of age) were purchased from SLC Japan (Yokohama, Japan) and 5-week-old male and female GPR14 KO mice were purchased from Charles River Japan (Tokyo, Japan). GPR14 KO mice were custom-generated and genotyped by Charles River Japan. Standard chow (FR-2; Funabashi Farm Co., Ltd., Chiba, Japan) and tap water were provided *ad libitum* to all mice under conditions of controlled temperature (23 ± 2 °C), humidity (55 ± 10%), and a 12-h light/dark cycle (lights on from 7:00 AM to 7:00 PM). All experimental procedures were performed in accordance with the guidelines of the Institutional Animal Care and Use Committee of Daiichi Sankyo Co., Ltd. The investigation conformed to the Guide for the Care and Use of Laboratory Animals, 8^th^ edition, updated by the U.S. National Research Council Committee in 2011.

### 2.2 Test compound

**Figure 1:**
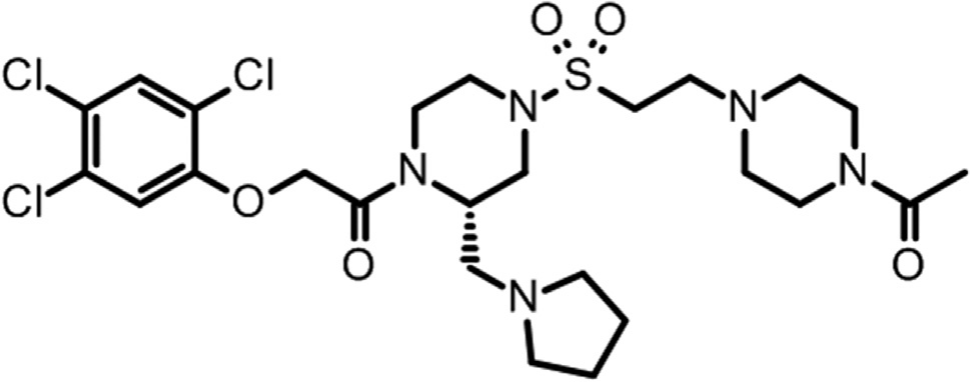
Chemical structure of DS37001789.

DS37001789 was synthesized at our laboratory. Its chemical structure is shown in Figure 1.

### 2.3 Transverse aortic constriction in mice

At the age of 8 weeks, the C57BL/6 N mice and GPR14 KO mice were anesthetized with isoflurane and their respiration was artificially controlled on a heating pad. Transverse aortic constriction (TAC) was performed as previously described [17]. Briefly, aortic constriction was performed by tying a 7-0 polypropylene suture around a 27-gauge needle placed on the aortic arch, which was then rapidly removed after ligation. Sham-operated mice underwent the same surgical operation without aortic constriction. Cardiac function was measured by echocardiography at 1 week after TAC and the mice were randomly divided into three groups by the echocardiographic parameters. The mice were fed a standard diet (FR-2; Funabashi Farms) containing 0.06% or 0.2% DS37001789 for 4 or 12 weeks.

### 2.4 Echocardiographic study

The echocardiographic study was performed 1 and 4 weeks after TAC or sham surgery in conscious mice. Echocardiography of the left ventricle (LV) was performed using an echocardiogram equipped with a 15 MHz linear probe (GE Healthcare, Milwaukee, WT). LV end-diastoric diameter (LVDd) and LV end-systolic diameter (LVDs) were obtained from M-mode. LV ejection fraction (EF) was calculated [18].

### 2.5 In vivo hemodynamics

LV function was assessed by pressure-volume (PV) analysis, as described previously [19]. Briefly, TAC mice were anesthetized, the chest was opened, and a miniature pressure-volume catheter was introduced (SPR-839 PV; Millar Instrument, Inc., Houston, TX) via the LV apex. The PV signal was recorded using the LabChart system (AD Instruments, Sydney, Australia).

### 2.6 RNA isolation and TaqMan real-time quantitative polymerase chain reaction (PCR)

mRNA levels were quantified in fresh-frozen LV. Total RNA was prepared using TRIzol reagent (Life Technologies, Carlsbad, CA) and the RNeasy Minikit (QIAGEN, Hilden, Germany). Reverse-transcription PCR was performed using SuperScript II Reverse Transcriptase (Life Technologies). Real-time PCR was performed using TaqMan Gene Expression Assays and primers for ANP, BNP, SERCA2a, Phospholamban, RCAN-1 COL1A2, and GAPDH mRNA (Thermo Fisher Scientific, Waltham, MA). The mRNA levels of each gene were normalized to the GAPDH level. Results are expressed as fold changes from the mRNA expression in the sham-operated group.

### 2.7 Western blotting

Protein levels of GPR14 and GAPDH were assessed in fresh frozen LV and rat isolated cardiomyocyte. Tissue and cells homogenized in lysis buffer (Cell Signaling Technology Inc., Danvers, MA) containing 1 mM phenylmethylsulfonyl fluoride (Sigma-Aldrich Co., LLC., St. Louis, MO) was centrifuged and protein was quantified by the bicinchoninic acid assay (Thermo Fisher Scientific); NuPAGE lithium dodecyl sulfate sample buffer (Thermo Fisher Scientific) was added and lysates were electrophoresed on NuPAGE 4%–12% Bis-Tris polyacrylamide gels (Thermo Fisher Scientific). Proteins were transferred to polyvinylidene difluoride membranes and incubated with primary antibodies, anti-GPR14 (1:200; Santa Cruz Biotechnology, Inc., Dallas, TX), and glyceraldehyde-3-phosphate dehydrogenase (GAPDH; 1:10,000; Cell Signaling Technology Inc.), followed by horseradish peroxidase-conjugated secondary antibodies (goat anti-mouse IgG1; Santa Cruz Biotechnology). Each sample was detected using ECL Advanced Western blotting detection kit (GE Healthcare UK Ltd., Little Chalfont, UK) and a chemiluminescence system. Band intensity was quantified using ImageJ software.

### 2.8 Rat isolated cardiomyocyte study

Cardiomyocytes were isolated from the heart of Sparague-Dawley rats (9 weeks of age). The heart was removed from the rat and placed the heart on the cannula of the Langendorff perfusion system. The heart was perfused with the Ca^2+^ free perfusion buffer (241 mM NaCl, 29.4 mM KCl, 1.2 mM KH_2_PO_4_, 1.2 mM Na_2_HPO_4_, 2.4 mM MgSO_4_, 20 mM HEPES, 9.2 mM NaHCO_3_, 5.6 mM D-glucose, 9.9 mM BDM, 29.9 mM Taurine), then switched to perfusion buffer containing 1 mg/ml type II collagenase (Worthington industries, Columbus, OH). The heart was placed in perfusion buffer containing 10% FBS (Thermo Fisher Scientific) and minced. The isolated cardiomyocyte were washed in tyroad buffer (1 mM CaCl_2_, 140 mM NaCl, 5 mM KCl, 10 mM HEPES, 1 mM MgCl2, 5.6 mM D-glucose) and incubated with DS37001789 for respective times.

### 2.9 Statistical analysis

All data in this study are expressed as mean ± S.E.M. Comparisons among groups were performed by Dunnett's test and *P <* 0.05 was considered to be statistically significant. Comparisons among groups in the echocardiography study were performed by two-way ANOVA and *P <* 0.05 was considered to be statistically significant. SAS System Release (SAS Institute Inc., Tokyo, Japan) was used to test significance.

## 3 Results

### 3.1 Effects of DS37001789 on cardiac hypertrophy and cardiac function after pressure overload in mice

**Figure 2:**
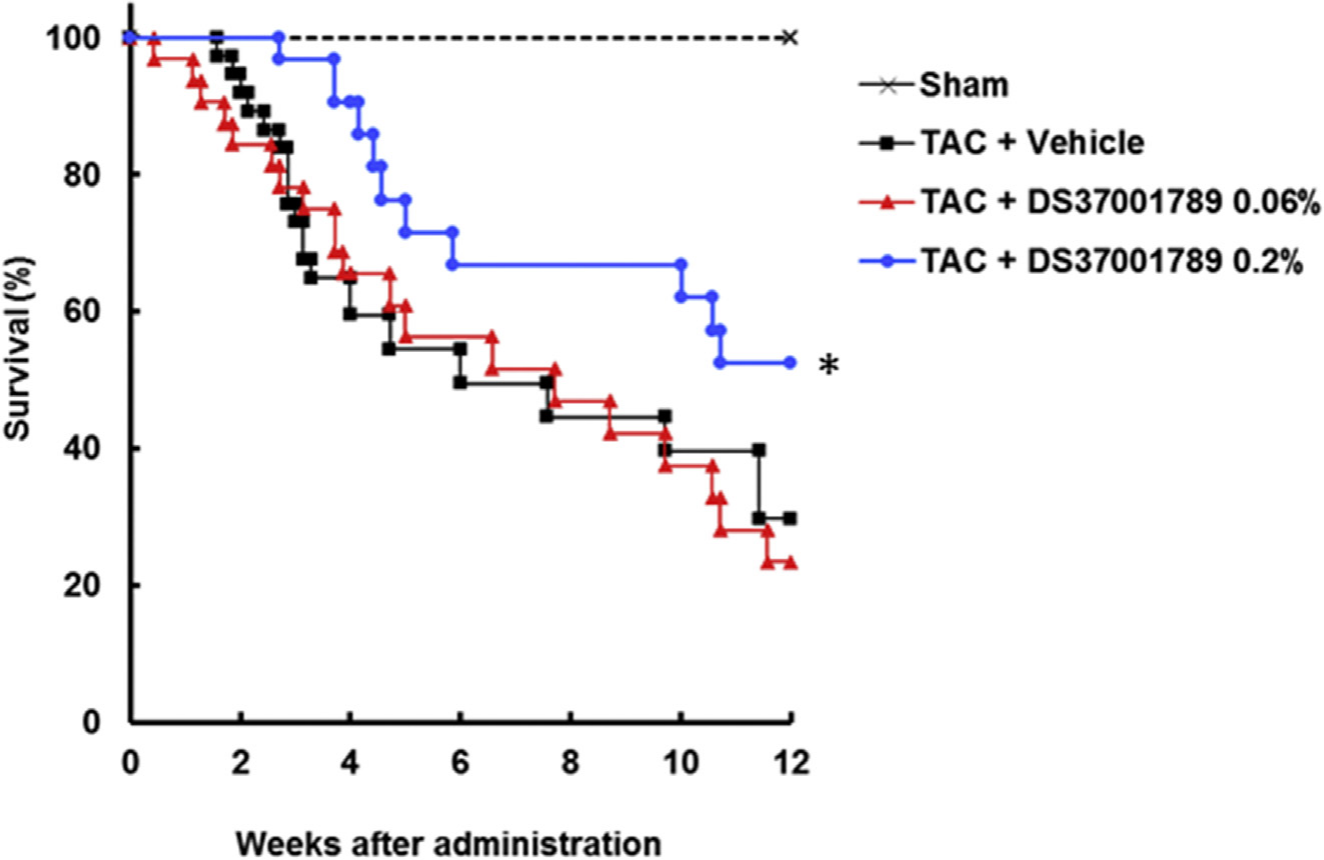
Effect of DS37001789 on mortality in pressure-overload mice. Kaplan–Meier survival plot of 12-week-old mice after the transverse aortic constriction (TAC) surgery. The survival rates of vehicle-treated sham-operated mice (Sham, n = 5), vehicle-treated TAC mice (Vehicle, n = 37), 0.06% DS37001789-treated TAC mice (DS37001789 0.06%, n = 32), and 0.2% DS37001789-treated TAC mice (DS37001789 0.2%, n = 32) are shown. *P < 0.05, significantly different from the vehicle-treated control group.

**Figure 3:**
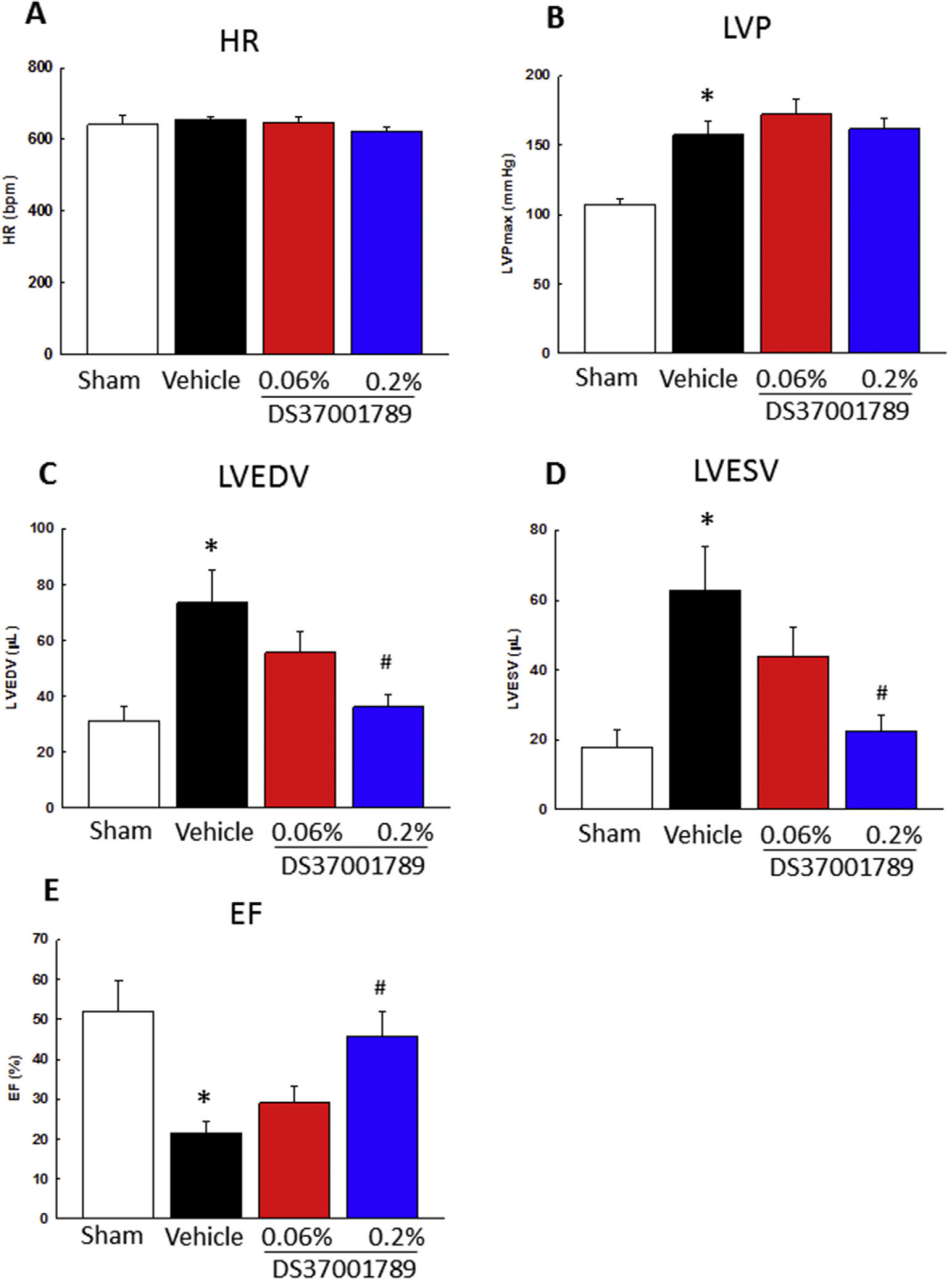
Effects of DS37001789 on hemodynamic parameters in pressure-overload mice. (A) Hemodynamic parameters of heart rate (HR), (B) left ventricular pressure (LVP), (C) left ventricular end-diastolic volume (LVEDV), (D) left ventricular end-systolic volume (LVESV), and (E) ejection fraction (EF) after 4-week administration of vehicle or DS37001789. Vehicle-treated sham-operated mice (Sham, n = 4), vehicle-treated transverse aortic constriction-induced pressure overload mice (TAC) (Vehicle, n = 4), 0.06% DS37001789-treated TAC mice (n = 4), and 0.2% DS37001789-treated TAC mice (n = 4) were measured. Data are expressed as mean ± S.E.M. *P < 0.05, significantly different from the sham-operated mice group; ^#^*P* < 0.05, significantly different from the TAC vehicle-treated control group.

**Figure 4:**
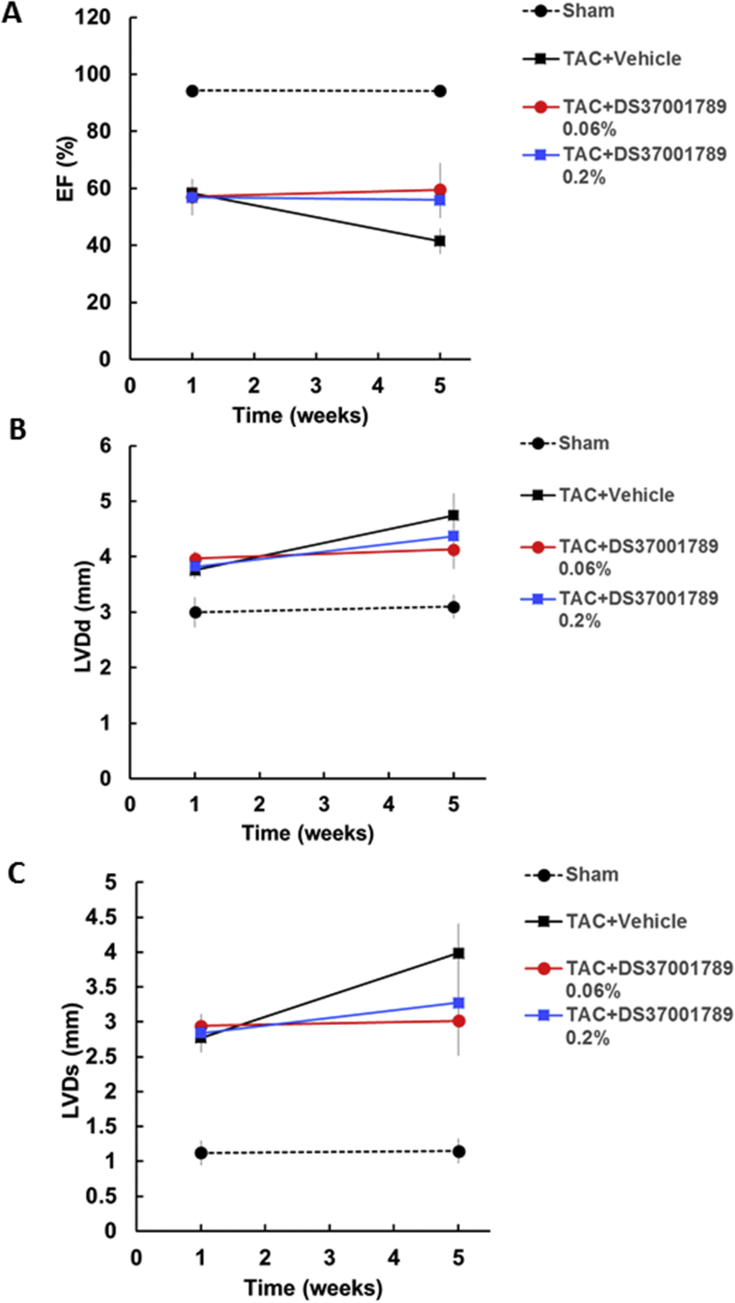
Effects of DS37001789 on echocardiographic parameters in pressure-overload mice. Echocardiographic parameters of (A) ejection fractions (EFs), (B) left ventricular end-diastric diameter (LVDd), and (C) left ventricular end-systolic diameter (LVDs) 1 week after the transverse aortic constriction (TAC) surgery and after 4 weeks of administration of vehicle or DS37001789. Vehicle-treated sham-operated mice (Sham, n = 3), vehicle-treated transverse aortic constriction-induced pressure-overload mice (TAC) (Vehicle, n = 11), 0.06% DS37001789-treated TAC mice (n = 7), and 0.2% DS37001789-treated TAC mice (n = 10) were measured. Data are expressed as mean ± S.E.M.

**Figure 5:**
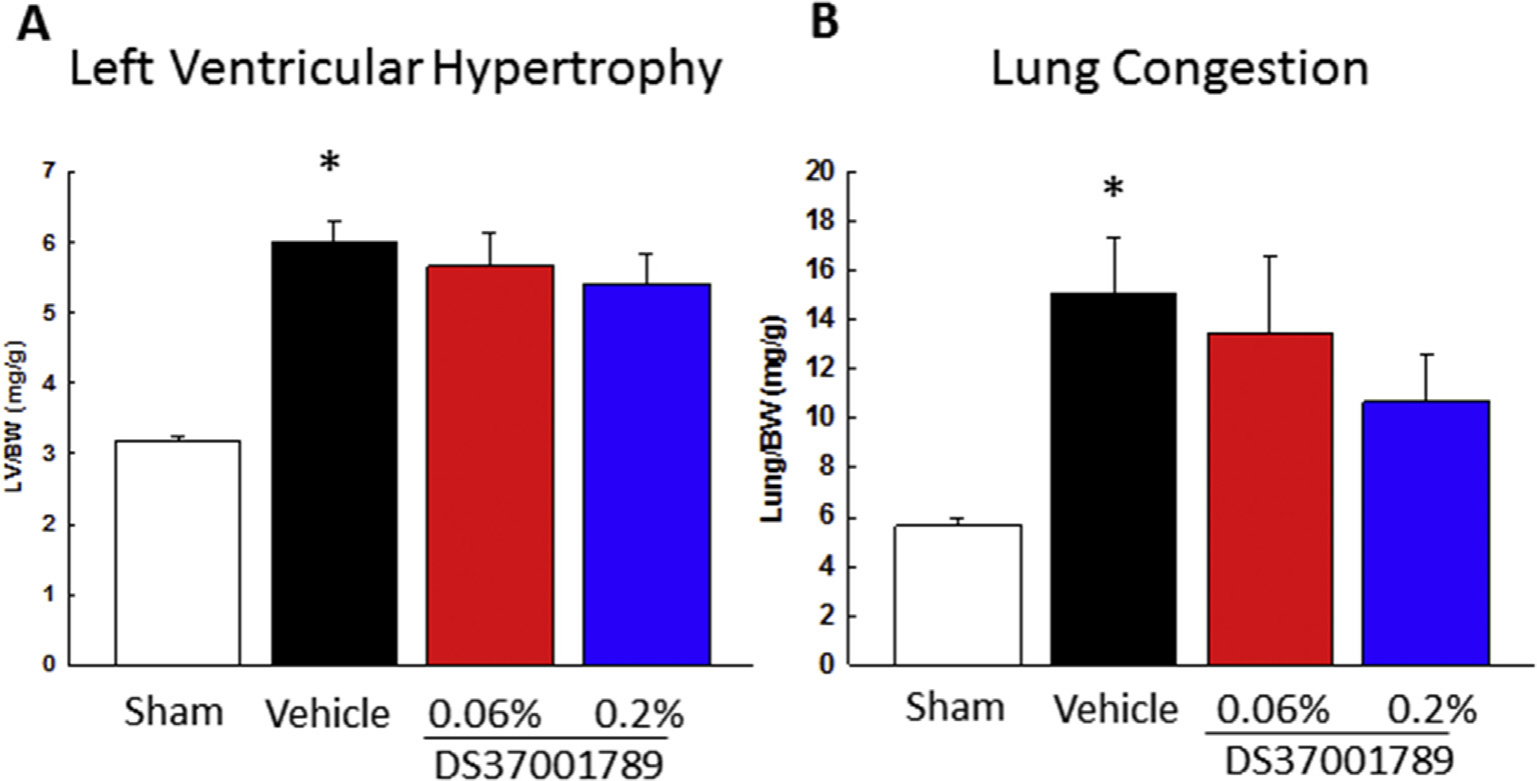
Effects of DS37001789 on tissue weight in pressure-overload mice. (A) Left ventricular weight/body weight, and (B) lung weight/body weight after 4 weeks of administration in pressure-overload mice. Vehicle-treated sham-operated mice (Sham, n = 8), vehicle-treated transverse aortic constriction-induced pressure-overload mice (TAC) (Vehicle, n = 15), 0.06% DS37001789-treated TAC mice (n = 10), and 0.2% DS37001789-treated TAC mice (n = 10) were measured. Data are expressed as mean ± S.E.M. *P < 0.05, significantly different from the sham-operated mouse group.

To determine the effects of DS37001789 against mortality, cardiac dysfunction, and cardiac hypertrophy, DS37001789 was orally administered to a TAC model. This is a well-established model of pressure-overload-induced cardiac hypertrophy and cardiac dysfunction mimicking human heart failure [20]. We evaluated mortality for 12 weeks and cardiac hypertrophy and function after 4 weeks of DS37001789 administration. There were no significant differences in growth rate of BW in either sham or TAC animals (data not shown). The group treated with a low dose of DS37001789 (0.06%) did not exhibit a significantly ameliorated survival rate compared with vehicle-treated TAC mice. On the other hand, the group treated with a high dose of DS37001789 (0.2%) showed significant amelioration of mortality compared with vehicle-treated TAC mice in Kaplan–Meier analyses (Figure 2, *P* < 0.05). To determine whether DS37001789 would affect cardiac function, invasive PV-loop analysis was performed on TAC mice after 4 weeks of DS37001789 administration (Figure 3). LV peak systolic pressure (LVP) and heart rate (HR) were similar in vehicle-treated TAC mice and DS37001789-treated TAC mice. Compared with sham-operated mice, vehicle-treated TAC mice exhibited significant increases in LV end-diastolic volume (LVEDV) (vehicle-treated sham vs. vehicle-treated TAC: 31.2 ± 5.4 μL vs. 73.5 ± 11.5 μL, *P* < 0.05) and LV end-systolic volume (LVESV) (vehicle-treated sham vs. vehicle-treated TAC: 17.9 ± 4.8 μL vs. 62.8 ± 12.4 μL, *P* < 0.05), and a significant decrease in ejection fraction (EF) (vehicle-treated sham vs. vehicle-treated TAC: 52.0 ± 7.5% vs. 21.6 ± 2.7%, *P* < 0.05). Compared with those in vehicle-treated TAC mice, the administration of 0.2% DS37001789 significantly ameliorated LVEDV (36.2 ± 4.4 μL, *P* < 0.05), LVESV (22.4 ± 4.5 μL, *P* < 0.05), and EF (45.9 ± 6.2%, *P* < 0.05). DS37001789 also tended to improve cardiac function and cavity enlargement, as revealed by echocardiography, although there were no significant differences between DS37001789- and vehicle-treated TAC mice (Figure 4). As identified by measurement of the ratio of left ventricular weight to body weight (LV/BW), TAC was sufficient to induce steady hypertrophy compared with that in sham-operated mice (vehicle-treated sham vs. vehicle-treated TAC: 3.2 ± 0.1 mg/g vs. 6.0 ± 0.3 mg/g, *P* < 0.05) (Figure 5). As identified by measurement of the ratio of lung weight to body weight (lung/BW), TAC also significantly increased lung congestion (vehicle-treated sham vs. vehicle-treated TAC: 5.7 ± 0.3 mg/g vs. 15.1 ± 2.3 mg/g, *P* < 0.05) (Figure 5). DS37001789 did not ameliorate LV/BW (5.7 ± 0.5 mg/g for 0.06%, 5.4 ± 0.4 mg/g for 0.2% DS37001789; *P* > 0.05) and lung/BW in the TAC-operated groups (13.5 ± 3.1 mg/g for 0.06%, 10.7 ± 2.0 mg/g for 0.2% DS37001789; *P* > 0.05).

**Figure 6:**
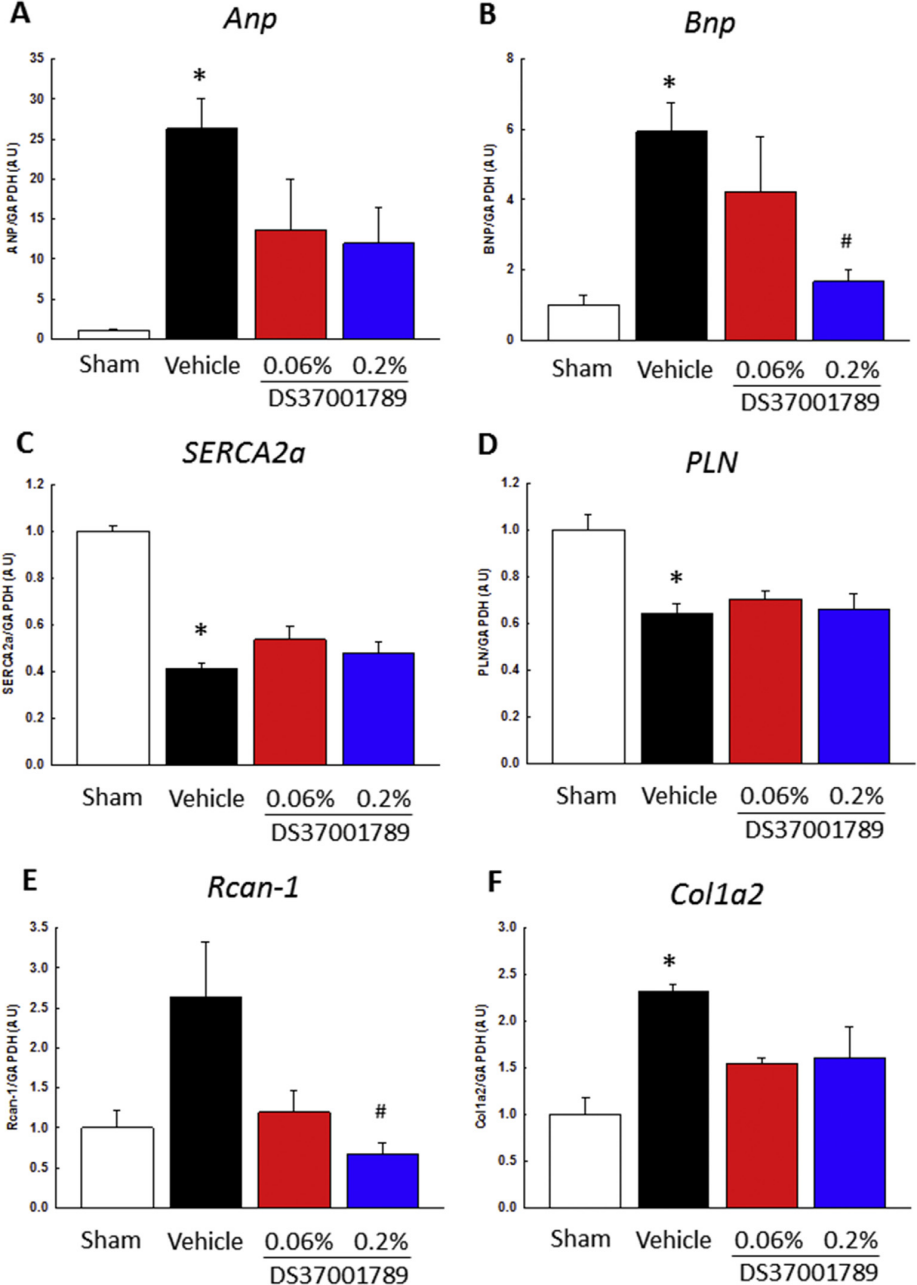
Effects of DS37001789 on mRNA expression in pressure-overload mice. (A) Atrial natriuretic peptide (ANP), (B) brain natriuretic peptide (BNP), (C) sarco/endoplasmic reticulum Ca^2+^-ATPase (SERCA2a), (D) phospholamban (PLN), (E) regulator of calcineurin 1 (RCAN-1), and (F) collagen type I alpha 2 (COL1A2) expression after 4 weeks of administration in pressure-overload mice. mRNA expression levels of *ANP*, *BNP*, *SERCA2a*, *PLB*, *RCAN-1*, and *COL1A2* normalized to *Gapdh* levels in vehicle-treated sham-operated mice (Sham, n = 3), vehicle-treated transverse aortic constriction-induced pressure-overload mice (TAC) (Vehicle, n = 4), 0.06% DS37001789-treated TAC mice (n = 3), and 0.2% DS37001789-treated TAC mice (n = 3) were measured. Data are expressed as mean ± S.E.M. *P < 0.05, significantly different from the sham-operated mouse group; ^#^*P* < 0.05, significantly different from the TAC vehicle-treated control group.

To determine whether DS37001789 had an anti-hypertrophic effect in TAC-induced mice after 4 weeks of administration, we measured the mRNA expression of regulator of calcineurin 1 (*Rcan-1*), which is a target gene of the calcineurin/NFAT pathway, and the mRNA expression of atrial natriuretic peptide (*ANP*) and brain natriuretic peptide (*BNP*), which are sensitive markers of heart failure and hypertrophy (Figure 6). The *Rcan-*1 mRNA expression in TAC left ventricles was about 2.6 ± 0.7-fold higher than that in sham-operated left ventricles (*P* > 0.05). The *Rcan-*1 mRNA expression upon the administration of 0.2% DS37001789 did significantly decrease compared with that in vehicle-treated TAC mice (0.7 ± 0.2, *P* < 0.05). The *ANP* mRNA expression in TAC left ventricles was about 26.3 ± 3.8-fold higher than that in sham-operated left ventricles (*P* < 0.05). The *ANP* mRNA expression upon the administration of 0.2% DS37001789 did not significantly decrease compared with that in vehicle-treated TAC mice, although there was a tendency for a decrease (11.9 ± 4.5, *P* < 0.1). On the other hand, the *BNP* mRNA expression in TAC left ventricles was about 5.9 ± 0.8-fold higher than that in sham-operated left ventricles (*P* < 0.05). The *BNP* mRNA expression upon the administration of 0.2% DS37001789 did significantly decrease compared with that of vehicle-treated TAC mice (1.7 ± 0.4, *P* < 0.05). To analyze the mechanism of the improvement of cardiac function by DS37001789, the mRNA expression levels of sarco/endoplasmic reticulum Ca^2+^-ATPase (*SERCA2a*) and phospholamban (*PLN*) were measured, which are calcium regulatory markers of HF (Figure 6). The *SERCA2a* mRNA expression in TAC left ventricles was 0.41 ± 0.03-fold that of sham-operated left ventricles (*P* < 0.05). The *SERCA2a* mRNA expression with 0.2% DS37001789 did not significantly increase compared with that of vehicle-treated TAC mice (0.48 ± 0.04, *P* > 0.05). The *PLN* mRNA expression in TAC left ventricles was 0.64 ± 0.04-fold that in sham-operated left ventricles (*P* < 0.05). The *PLN* mRNA expression with 0.2% DS37001789 did not significantly increase compared with that in vehicle-treated TAC mice (0.7 ± 0.1, *P* > 0.05). There was also no significant improvement in calcium regulatory markers. Next, to determine the effect of DS37001789 on fibrosis, the *Col1a2* mRNA expression was measured (Figure 6). The *Col1a2* mRNA expression in TAC left ventricles was 2.3 ± 0.1-fold higher than that in sham-operated left ventricles (*P* < 0.05). The *Col1a2* mRNA expression with 0.2% DS37001789 did not significantly decrease compared with that in vehicle-treated TAC mice (1.6 ± 0.3, *P* > 0.05). From these results, no anti-fibrotic effect or improvement of cardiac function markers was achieved by DS37001789 in TAC-induced mice.

**Figure 7:**
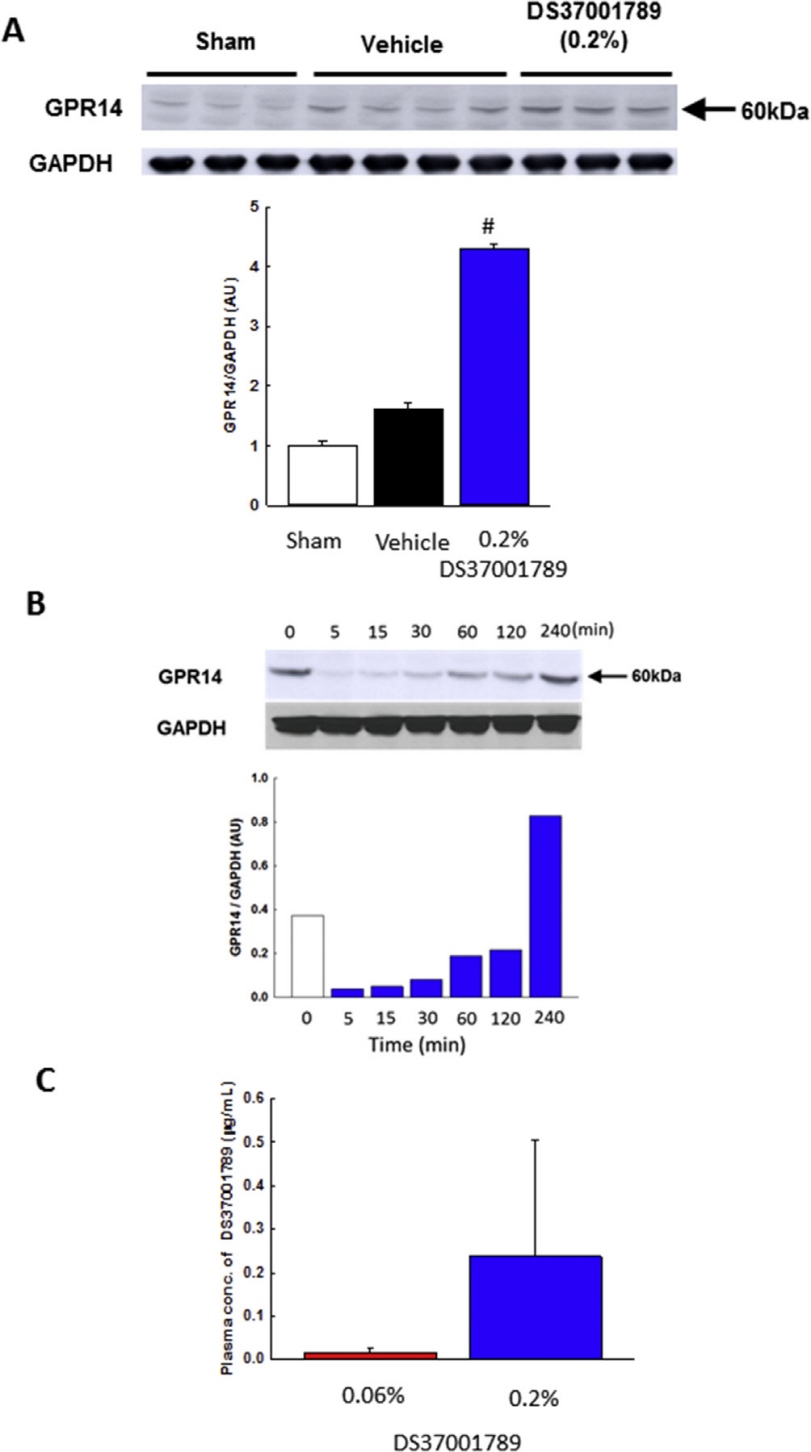
Effect of cardiac GPR14 expression on DS37001789 and plasma concentrations of DS37001789 after 4 weeks of administration in pressure-overload mice. (A) GPR14 expression in left ventricle by western blot analysis. GPR14 expression levels normalized to GAPDH levels in vehicle-treated sham-operated mice (Sham, n = 3), vehicle-treated transverse aortic constriction-induced pressure-overload mice (TAC) (Vehicle, n = 4), and 0.2% DS37001789-treated TAC mice (n = 3) were measured. Data are expressed as mean ± S.E.M. (B) GPR14 expression in rat isolated cardiomyocyte by western blot analysis. GPR14 expression levels normalized to GAPDH levels. (C) Plasma compound concentrations in 0.06% DS37001789-treated TAC mice (n = 6) and 0.2% DS37001789-treated TAC mice (n = 10) were measured. Data are expressed as mean ± S.E.M. The full, uncropped versions of the blots can be found as supplementary materials.

To determine whether DS37001789 affected GPR14 expression in TAC-induced mice after 4 weeks of administration, we measured the GPR14 protein expression in TAC left ventricles (Figure 7A). GPR14 expression increased 61% in vehicle-treated TAC left ventricles compared with that in sham-operated left ventricles, but the difference was not significant. However, GPR14 expression was significantly upregulated in 0.2% DS37001789-treated TAC left ventricles (vehicle-treated TAC vs. 0.2% DS37001789-treated TAC: 1.6 ± 0.1 vs. 4.3 ± 0.1, *P* < 0.05). We evaluated whether or not the upregulated GPR14 expression effect of DS37001789 in TAC mice was direct action using rat isolated cardiomyocyte. DS37001789 (600nM) upregulated the GPR14 expression in time-dependent manner and the GPR14 expression at 240 min after treatment of DS37001789 was markedly increased compared with that in DS37001789-untreated cardiomyocyte (Figure 7B). These results suggest that DS37001789 directly acts on GPR14 receptor to prevent pressure-overload-induced cardiac dysfunction. Next, to evaluate the relationship between pharmacological effects and plasma compound concentrations, plasma concentrations of DS37001789 were measured at 4 weeks after DS37001789 administration. Dose-dependent increases in plasma DS37001789 concentration were seen, and the DS37001789 concentrations were 1.67 and 23.2 ng/ml upon treatment with 0.06% and 0.2% DS37001789, respectively (Figure 7C).

### 3.2 Effect of GPR14 deletion on cardiac function after pressure overload in mice

**Figure 8:**
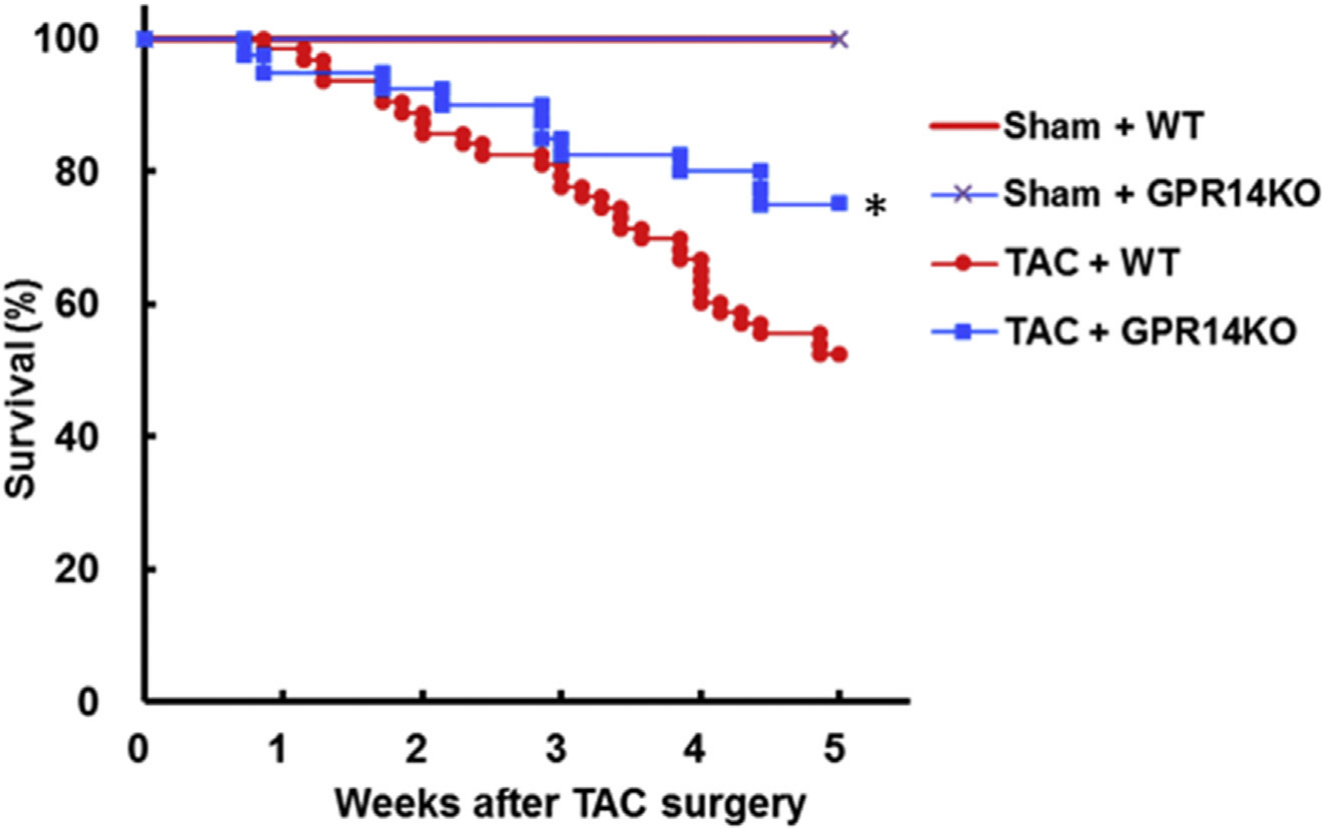
Mortality after pressure overload in GPR14 knockout (KO) mice. Kaplan–Meier survival plot of 5-week-old mice after the transverse aortic constriction (TAC) surgery. The survival rates of sham-operated wild-type (WT) mice (Sham + WT, n = 14), sham-operated GPR14 KO mice (Sham + GPR14 KO, n = 13), TAC-operated WT mice (TAC + WT, n = 63), and TAC-operated GPR14 KO mice (TAC + GPR14KO, n = 40) are shown. *P < 0.05, significantly different from the TAC WT group.

**Figure 9:**
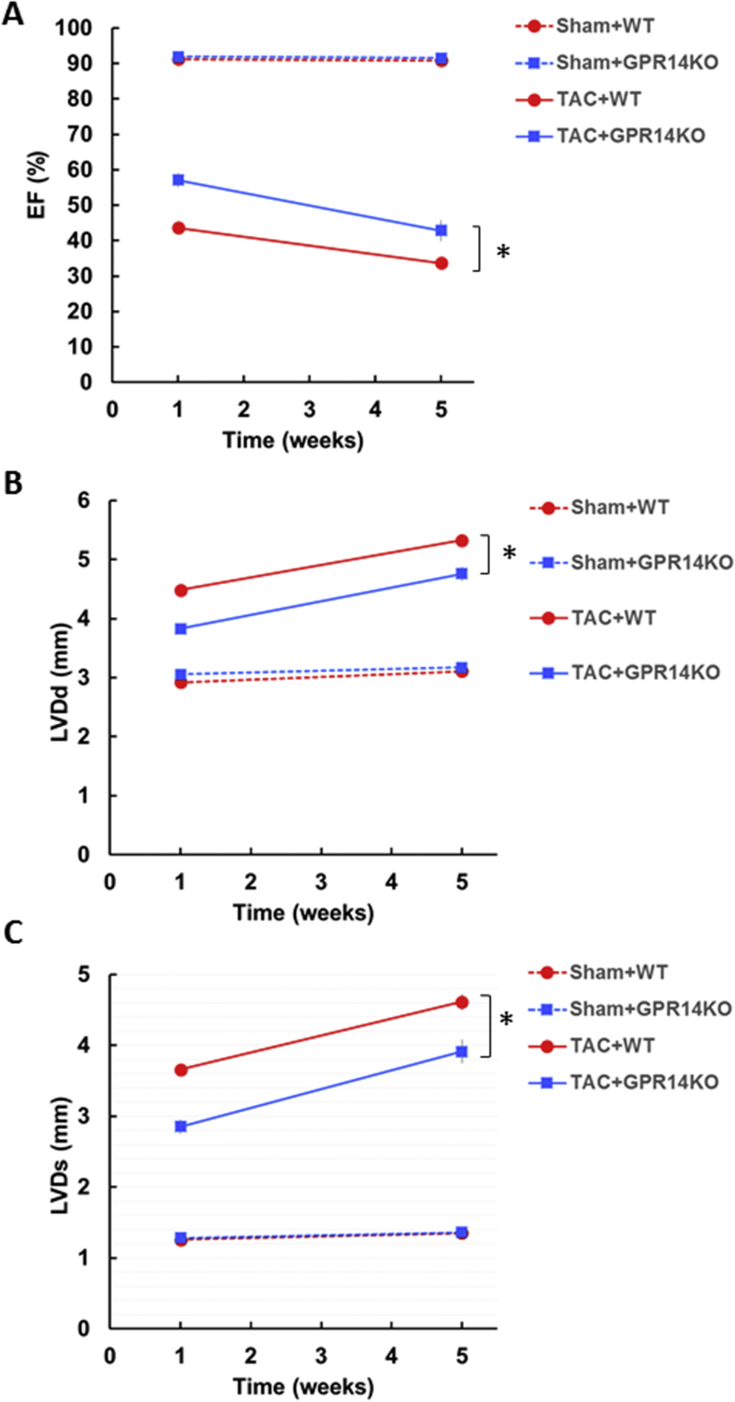
Effect of GPR14 knockout (KO) on echocardiographic parameters in pressure-overload mice. Echocardiographic parameters of (A) ejection fractions (EFs), (B) left ventricular end-diastoric diameter (LVDd), and (C) left ventricular end-systolic diameter (LVDs) at 1 and 4 weeks after the transverse aortic constriction (TAC) surgery. The results of sham-operated WT mice (Sham + WT, n = 10), sham-operated GPR14 KO mice (Sham + GPR14KO, n = 8), TAC-operated WT mice (TAC + WT, n = 35), and TAC-operated GPR14KO mice (TAC + GPR14KO, n = 30) are shown. Data are expressed as mean ± S.E.M. *P < 0.05, significantly different from the TAC WT group.

**Figure 10:**
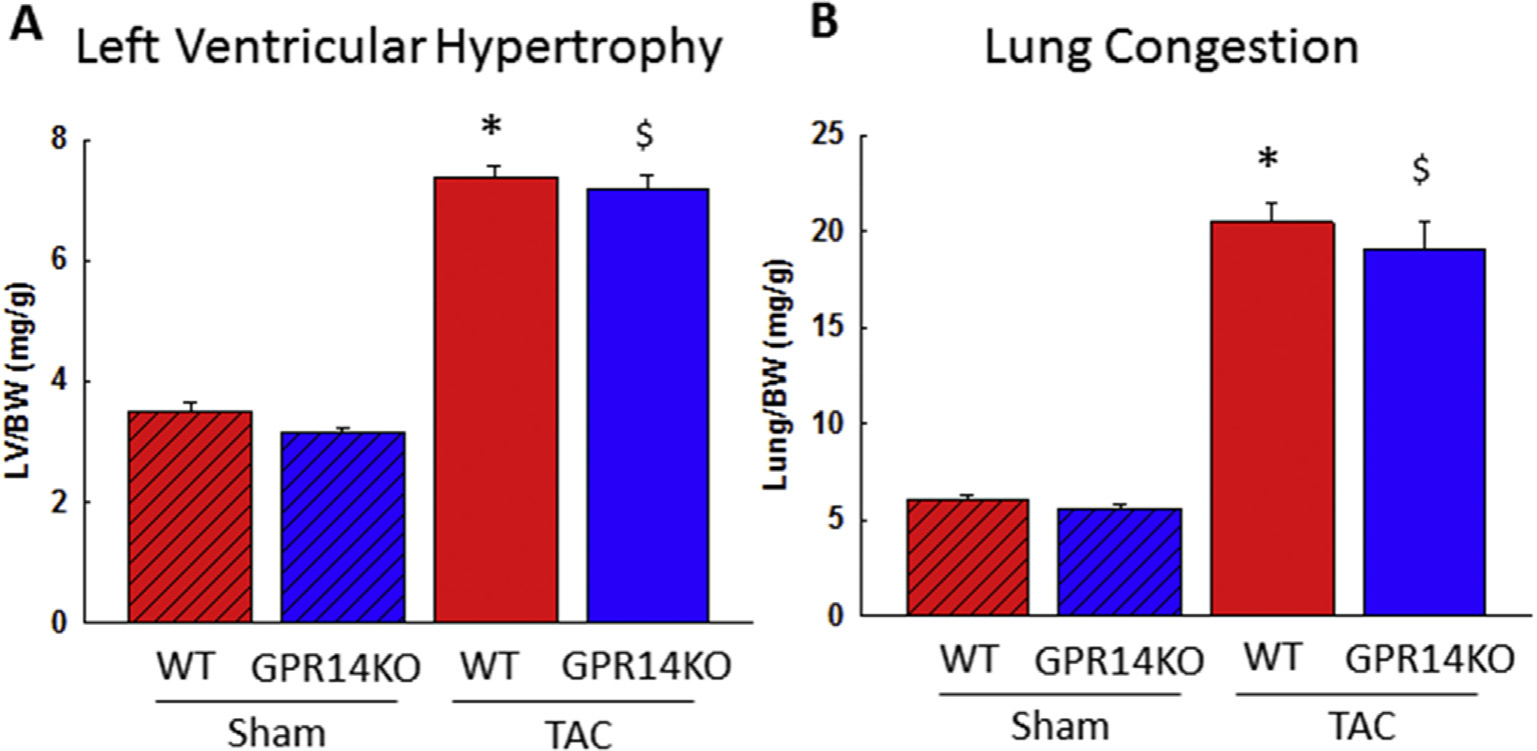
Effects of GPR14 knockout (KO) on tissue weight 4 weeks after the transverse aortic constriction (TAC) surgery. (A) Left ventricular weight/body weight and (B) lung weight/body. Results of sham-operated WT mice (n = 4), sham-operated GPR14 KO mice (n = 4), TAC-operated WT mice (n = 12), and TAC-operated GPR14 KO mice (n = 9) are shown. Data are expressed as mean ± S.E.M. *P < 0.05, significantly different from the sham WT group. ^$^*P* < 0.05, significantly different from the sham GPR14 KO group.

The effect of endogenous GPR14 deletion after TAC has not been reported previously. Therefore, we investigated the effect of this deletion on cardiac hypertrophy and function using a TAC model in mice. There was no difference in BW and food intake in either WT or GPR14 KO mice at 5 weeks after TAC surgery (data not shown). However, GPR14 KO TAC mice showed improved mortality compared with WT TAC mice in Kaplan–Meier analyses (*P* < 0.05, Figure 8). To determine the mechanisms involved in the amelioration of mortality in GPR14 KO TAC mice, echocardiographic studies were performed on TAC-induced mice at 1 and 4 weeks after TAC operation. In GPR14 KO TAC mice, EF (GPR14 KO TAC mice vs. WT TAC mice: 42.8 ± 1.5% vs. 33.6 ± 1.5%), LVDd (GPR14 KO TAC mice vs. WT TAC mice: 4.8 ± 0.1 mm vs. 5.3 ± 0.1 mm), and LVDs (GPR14 KO TAC mice vs. WT TAC mice: 3.9 ± 0.2 mm vs. 4.6 ± 0.1 mm) were significantly ameliorated from 1 week after TAC operation compared with those in WT TAC mice (*P* < 0.05, Figure 9), whereas LV hypertrophy was similar to that in WT mice (Figure 10). These results suggest that GPR14 plays an important role in determining the survival rate and cardiac function in heart failure.

## 4 Discussion

We recently reported that DS37001789, a highly potent U-II antagonist with a novel piperazine derivative structure [16], showed a strong inhibitory profile against [^125^I]U-II binding to GPR14 (IC_50_ 0.9 nM). In addition, the contraction of aortas isolated from rat and monkey was induced by U-II. Moreover, DS37001789 showed U-II-induced pressor response blockade in mice, when administered orally. These findings suggest that DS37001789 is an orally available GPR14 antagonist with small species differences in pharmacological efficacy. Moreover, our results clearly demonstrated that DS37001789 has greater potential against U-II-induced cardiovascular contraction than ACT-058362, which failed in a clinical study potentially due to its low potency [21].

In this study, we demonstrated that DS37001789 ameliorated mortality in pressure-overload mice with heart failure. Moreover, in GPR14 KO mice, the mortality was significantly ameliorated via improvements in cardiac cavity enlargement and cardiac function in pressure-overload mice with heart failure. This study is the first to demonstrate the effect of U-II/GPR14 system blockade on cardiac dysfunction.

The increased expression of U-II and GPR14 has been reported in heart failure patients and in a pressure-overload animal heart failure model [8, 10, 11, 22, 23]. These results demonstrate that the U-II/GPR14 system plays an important role in heart failure. However, previous studies showed that a non-peptide U-II antagonist, SB-657510, had inefficient effects on pressure-overload-induced cardiac hypertrophy and cardiac dysfunction [15]. On the other hand, although another non-peptide U-II antagonist, KR-36996, showed anti-hypertrophic effects and prevention of cardiac dysfunction in pressure-overload-induced cardiac hypertrophy models [14], that study did not clarify the effect on mortality in heart failure models. Accordingly, there is still no consensus about the effect of U-II antagonists on improving the prognosis in heart failure. Therefore, we evaluated the effect of DS37001789 on cardiac function and mortality in TAC mice to clarify the beneficial effect of U-II/GPR14 system blockade in heart failure. Our results showed that the oral administration of 0.2% DS37001789 by mixing it with food for 12 weeks significantly ameliorated mortality. To understand the mechanism of action behind this, we investigated the effects of 0.2% DS37001789 on cardiac function, and against hypertrophy and fibrosis. Pressure-volume loop analysis and RNA expression analysis of *Rcan-1*, *ANP*, and *BNP* as heart failure and hypertrophy markers; *Col1a2* as a fibrosis marker; and *SERCA2a* and *PLB* as calcium regulatory markers were performed after 4 weeks of administration of 0.2% DS37001789 in TAC mice. In the pressure-volume loop studies, compared with the findings in the vehicle-treated TAC group, 0.2% DS37001789 significantly ameliorated LVEDV, LVESV, and EF without changing LVP. Furthermore, in gene expression studies, 0.2% DS37001789 significantly decreased the levels of hypertrophy markers. However, 0.2% DS37001789 did not significantly ameliorate the LV/BW that deteriorated due to the TAC operation. DS37001789 also did not show anti-fibrotic effects on pressure overload in this study. These results suggest that its main mechanism of action in improving mortality is based on the improvement of cardiac function.

Next, we evaluated the effect of DS37001789 on cardiac GPR14 expression in TAC mice. The GPR14 expression of the TAC group treated with 0.2% DS37001789 tended to be upregulated compared with that in the vehicle-treated TAC group. In order to clarify whether or not DS37001789 directly contributes to the upregulation of GPR14 expression in the heart, we evaluated the effect on the isolated cardiomyocyte. The GPR14 expression in DS37001789-treated cardiomyocyte tended to be upregulated compared with that in DS37001789-untreated cardiomyocyte. This result indicates that the upregulation of GPR14 in TAC mice may be caused by direct action of DS37001789 and suggests that DS37001789 acts on GPR14 in TAC mice. Next, we evaluated the association between pharmacological effects and plasma compound concentrations in this study. We calculated the estimated plasma compound concentrations that exert pharmacological effects from the values of in vitro activity (IC_50_ 0.9 nM) and the level of the free form of DS37001789 in mouse plasma. The plasma protein-binding rate of DS37001789 in mice was 90.2% (data not shown). Therefore, the estimated plasma IC_50_ concentration was 0.56 ng/ml. DS37001789 exposure levels at doses of 0.06% and 0.2% administered for 4 weeks in the TAC mouse model were 3 and 42 times higher than the estimated plasma IC_50_ concentration, respectively. In a previous study, we described that DS37001789 orally administered at a concentration of 30 mg/kg significantly blocked U-II-induced pressor response in mice. The plasma concentration of DS37001789 was 25-fold higher than the estimated IC_50_ concentration. The drug concentration upon 0.2% DS37001789 treatment was high enough to block U-II-induced pressor response in mice. These results suggested that 0.2% DS37001789 would achieve a plasma concentration that is sufficient to block a physiological U-II/GPR14 response and significantly ameliorated the mortality in pressure-overload mice due to improvement in cardiac function.

Ventricular remodeling involves changing of the structure and shape of the heart in order to maintain it in response to hemodynamic load, and is observed after myocardial infarction or after chronic pressure and volume load [24]. Stopping or reversing its progression is often referred to as “reverse remodeling.” The concept of reverse remodeling is defined as a reduction in LV volume and improvement of function [25]. It is successfully achieved by inhibitors of the renin-angiotensin-aldosterone system, β-blockers, and device-related therapies such as cardiac resynchronization or the use of ventricular assist devices [26, 27, 28, 29]. In these clinical trials, it was proven that reverse remodeling can improve prognosis in heart failure patients [30]. In this study, DS37001789 significantly ameliorated the deteriorations in LVEDV, LVESV, and EF caused by pressure overload. From these findings, we speculated that the improvement of mortality rate by DS37001789 occurred via reverse remodeling, given the improvements of EF, LVEDV, and LVESV.

We also evaluated the effects on cardiac function and mortality improvement using TAC mice with endogenous GPR14 deletion in order to obtain more reliable evidence that the U-II/GPR14 system plays a crucial role in heart failure. The effects of the U-II/GPR14 system in heart failure using GPR14 KO mice have not been reported. Although TAC induced a high mortality rate based on cardiac hypertrophy and cardiac dysfunction in WT mice, in GPR14 KO mice, the mortality was significantly ameliorated. To understand the mechanism of action behind such an effect, we measured cardiac function by echocardiography. The findings revealed that GPR14 KO TAC mice had significantly improved cardiac cavity enlargement and cardiac function. These findings suggested that endogenous GPR14 blockade would be valid as a strategy for treating heart failure. In addition, study of GPR14KO TAC mice further demonstrated that the effect of ameliorating mortality by U-II/GPR14 system blockade involved the improvement of cardiac function. On the other hand, echocardiography results showed some differences between DS37001789 and endogenous GPR14 blockade. We consider that these differences might have been due to differences between birth defects and acquired suppression of GPR14.

There is a major limitation in this study, which should be addressed in future research. Specifically, although U-II/GPR14 system blockade had no significant anti-hypertrophic effect, cardiac function was markedly improved by it, which in turn led to ameliorated mortality. However, the molecular mechanisms behind this are unknown. Therefore, further research is needed to clarify the molecular mechanisms behind the improvement of cardiac function by U-II/GPR14 system blockade in heart failure.

Recently, increased expression of U-II and GPR14 has been identified in prostate cancer and bladder cancer tissues [31, 32]. In addition, the mRNA expression levels of U-II and GPR14 were found to be increased in various human tumor cells, including HeLa cervical cancer cells, BeWo choriocarcinoma cells, IMR-32 neuroblastoma cells, and VMRC-RCW human renal cell carcinoma cells [33, 34]. These findings suggest that U-II/GPR14 is involved in cancer growth. As DS37001789 is a highly potent U-II receptor antagonist, advancing research on its use as a pharmacological tool may reveal the possibility of using U-II receptor antagonists as anti-cancer agents. Further progress in research in this field is anticipated.

## 5 Conclusion

The present study demonstrates that DS37001789 ameliorated mortality via an improvement in cardiac dysfunction in mice with pressure-overload-induced heart failure. In addition, in GPR14-deficient mice with pressure-overload-induced heart failure, mortality was significantly ameliorated via improved cardiac cavity enlargement and cardiac function. From these findings, we suggest that U-II/GPR14 system blockade improves cardiac function in heart failure. The U-II/GPR14 system may thus be an attractive target for treating heart failure with pathological cardiac hypertrophy. Moreover, DS37001789 may be a novel therapeutic agent for treating heart failure for patients with pressure-overload conditions such as hypertension and aortic valve stenosis.

## Declarations

### Author contribution statement

M. Nishi: Performed the experiments; Analyzed and interpreted the data; Wrote the paper.

H. Tagawa: Performed the experiments; Analyzed and interpreted the data.

M. Ueno: Performed the experiments.

S. Marumoto: Contributed reagents, materials, analysis tools or data.

T. Nagayama: Conceived and designed the experiments; Performed the experiments; Analyzed and interpreted the data.

### Funding statement

This work was supported by Daiichi Sankyo Co., Ltd.

### Competing interest statement

The authors declare no conflict of interest.

### Additional information

No additional information is available for this paper.

## Appendix A Supplementary data

Figure7. A Figure7. A

Figure7. B Figure7. B
