# Supplementary material for: The urotensin II receptor antagonist DS37001789 ameliorates mortality in pressure-overload mice with heart failure
Source: Heliyon. 2020 Feb 3;6(2):e03352. doi: 10.1016/j.heliyon.2020.e03352 (PMC7005433; doi:10.1016/j.heliyon.2020.e03352)
Supplement: Figure7. A [file mmc1.pptx]

## Slide 1
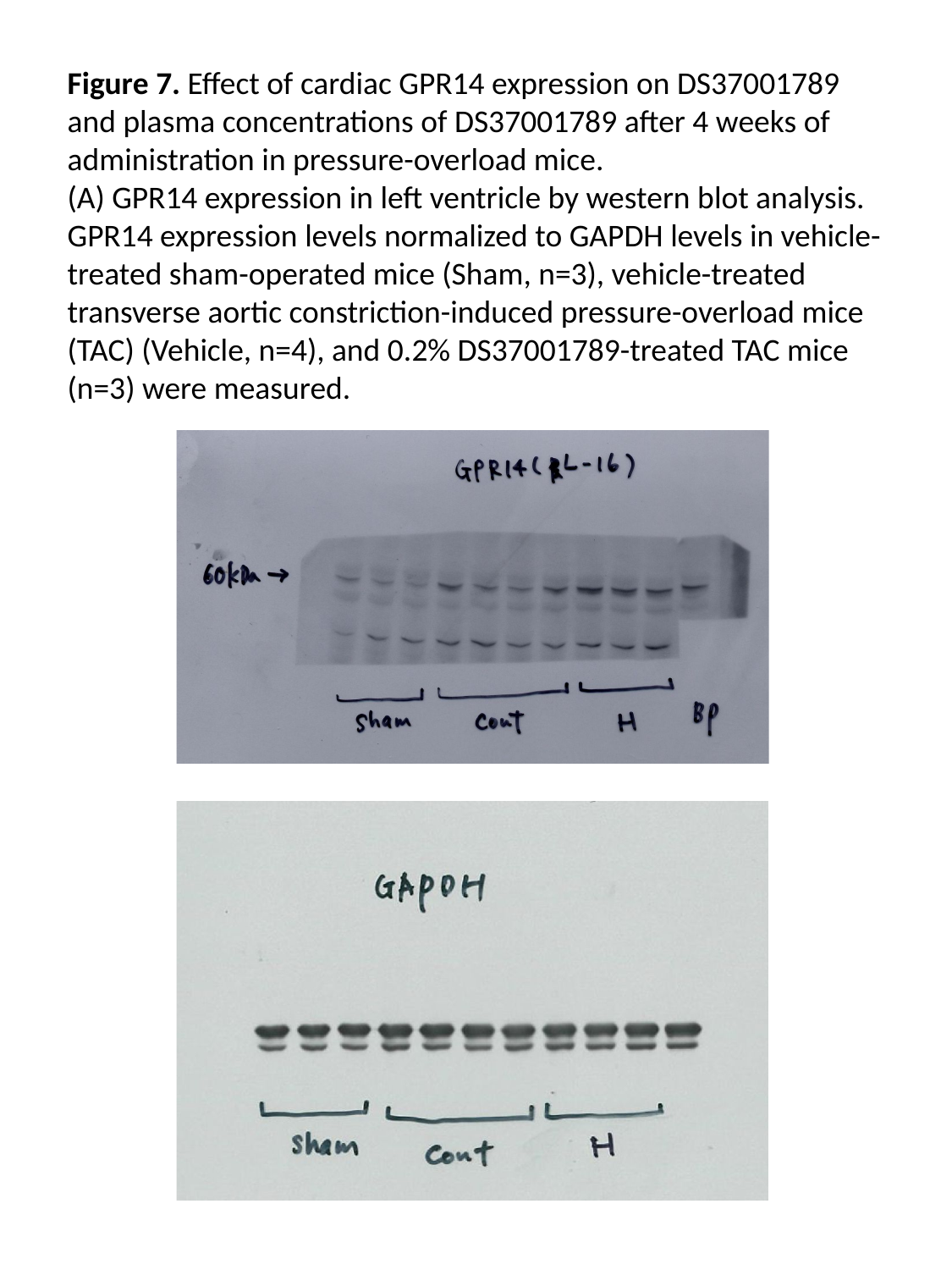

Figure 7. Effect of cardiac GPR14 expression on DS37001789 and plasma concentrations of DS37001789 after 4 weeks of administration in pressure-overload mice.
(A) GPR14 expression in left ventricle by western blot analysis. GPR14 expression levels normalized to GAPDH levels in vehicle-treated sham-operated mice (Sham, n=3), vehicle-treated transverse aortic constriction-induced pressure-overload mice (TAC) (Vehicle, n=4), and 0.2% DS37001789-treated TAC mice (n=3) were measured.
