# Supplementary material for: The urotensin II receptor antagonist DS37001789 ameliorates mortality in pressure-overload mice with heart failure
Source: Heliyon. 2020 Feb 3;6(2):e03352. doi: 10.1016/j.heliyon.2020.e03352 (PMC7005433; doi:10.1016/j.heliyon.2020.e03352)
Supplement: Figure7. B [file mmc2.pptx]

## Slide 1
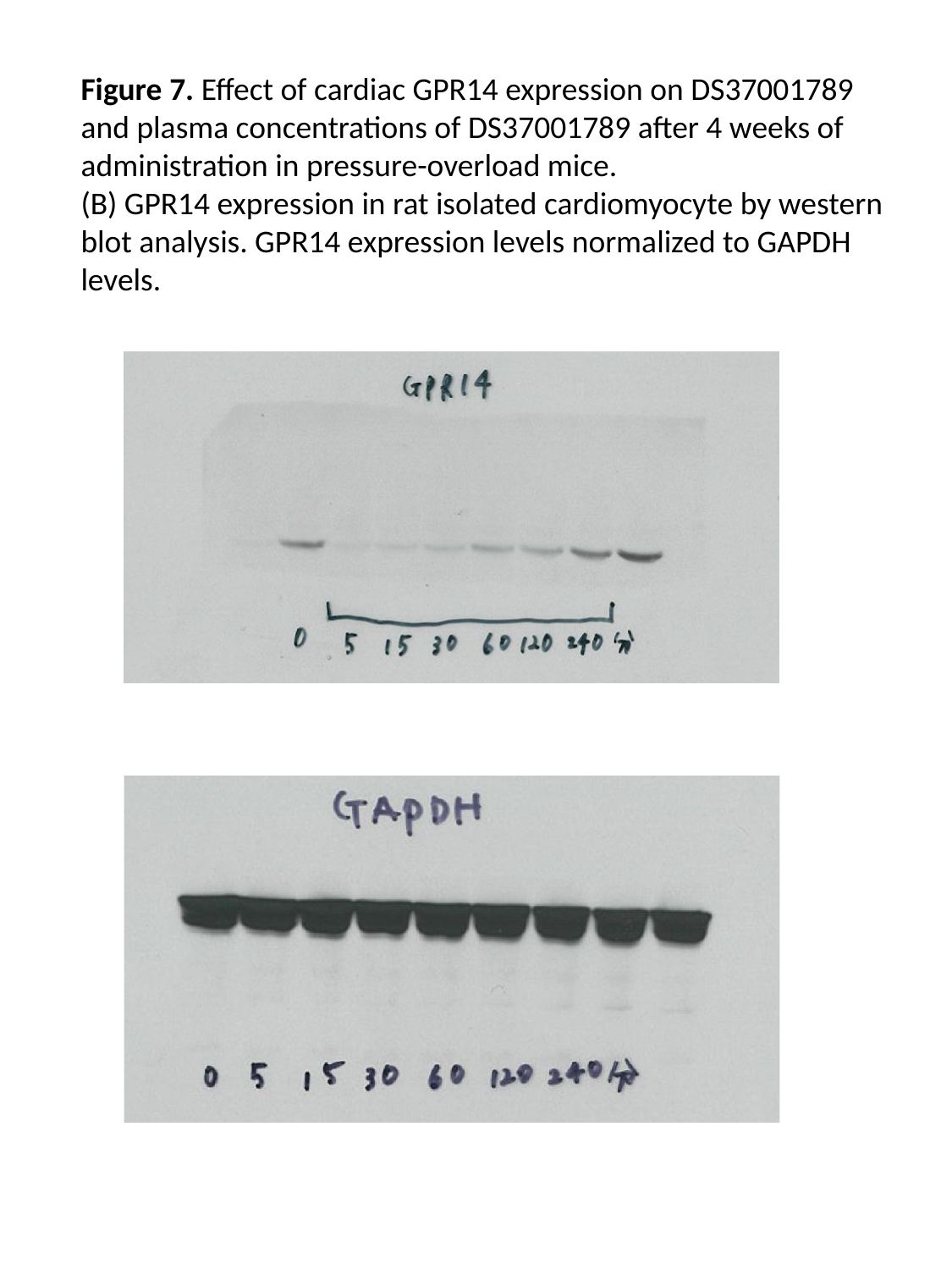

Figure 7. Effect of cardiac GPR14 expression on DS37001789 and plasma concentrations of DS37001789 after 4 weeks of administration in pressure-overload mice.
(B) GPR14 expression in rat isolated cardiomyocyte by western blot analysis. GPR14 expression levels normalized to GAPDH levels.
